# 3-(3-Meth­oxy­benzo­yl)-1,1-diphenyl­thio­urea

**DOI:** 10.1107/S1600536811025785

**Published:** 2011-07-09

**Authors:** Mohd Faizal Md Nasir, Ibrahim N. Hassan, Bohari M. Yamin, W. R. W Daud, Mohammad B. Kassim

**Affiliations:** a Fuel Cell Institute, University Kebangsaan Malaysia, 43600 Selangor, Malaysia; b School of Chemical Sciences & Food Technology, Faculty of Science & Technology, University Kebangsaan Malaysia, 43600 Selangor, Malaysia; c Department of Chemical and Process Engineering, Faculty of Engineering and Built Environment, University Kebangsaan Malaysia, 43600 Selangor, Malaysia; d School of Chemical Sciences & Food Technology, Faculty of Science & Technology, University Kebangsaan Malaysia, 43600 Selangor, Malaysia

## Abstract

The thiono and carbonyl groups in the title compound, C_21_H_18_N_2_O_2_S, adopt an *anti* disposition with respect to the central C—N bond. The diphenyl­amine rings are twisted relative to each other by a dihedral angle of 82.55 (10)°. The 3-meth­oxy­benzoyl fragment is twisted relative to one of the diphenyl­amine rings, forming a dihedral angle of 74.04 (9)°. In the crystal, pairs of inter­molecular N—H⋯S hydrogen bonds link the mol­ecules into centrosymmetric dimers, forming columns parallel to the *a* axis.

## Related literature

For related structures and background references, see: Al-abbasi *et al.* (2010 ▶); Al-abbasi & Kassim (2011 ▶); Md Nasir *et al.* (2011 ▶). For metal complexes of benzoyl­thio­ureas, see: Circu *et al.* (2009 ▶); Weiqun *et al.* (2005 ▶). For the synthetic procedure, see: Hassan *et al.* (2008 ▶). For a standard bond lengths, see: Allen *et al.* (1987 ▶).
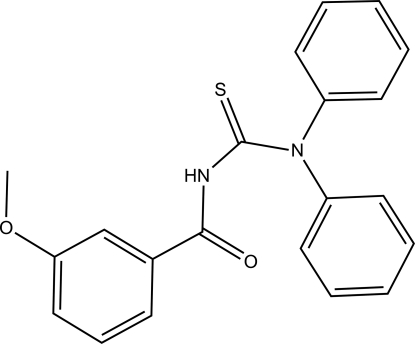


## Experimental

### 

#### Crystal data

C_21_H_18_N_2_O_2_S
*M* *_r_* = 362.43
Triclinic, 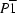

*a* = 6.056 (3) Å
*b* = 12.895 (7) Å
*c* = 13.344 (7) Å
α = 112.796 (9)°
β = 100.336 (10)°
γ = 97.951 (10)°
*V* = 920.0 (8) Å^3^
*Z* = 2
Mo *K*α radiation
μ = 0.19 mm^−1^
*T* = 298 K
0.52 × 0.23 × 0.03 mm

#### Data collection

Bruker SMART APEX CCD area-detector diffractometer
Absorption correction: multi-scan (*SADABS*; Bruker, 2000 ▶) *T*_min_ = 0.906, *T*_max_ = 0.994
10189 measured reflections
3612 independent reflections
2663 reflections with *I* > 2σ(*I*)
*R*_int_ = 0.029

#### Refinement

*R*[*F* ^2^ > 2σ(*F* ^2^)] = 0.038
*wR*(*F* ^2^) = 0.110
*S* = 1.04
3612 reflections
240 parameters
H atoms treated by a mixture of independent and constrained refinement
Δρ_max_ = 0.17 e Å^−3^
Δρ_min_ = −0.27 e Å^−3^

### 

Data collection: *SMART* (Bruker, 2000 ▶); cell refinement: *SAINT* (Bruker, 2000 ▶); data reduction: *SAINT*; program(s) used to solve structure: *SHELXS97* (Sheldrick, 2008 ▶); program(s) used to refine structure: *SHELXL97* (Sheldrick, 2008 ▶); molecular graphics: *SHELXTL* (Sheldrick, 2008 ▶); software used to prepare material for publication: *SHELXTL* and *PLATON* (Spek, 2009 ▶).

## Supplementary Material

Crystal structure: contains datablock(s) I, global. DOI: 10.1107/S1600536811025785/jh2306sup1.cif

Structure factors: contains datablock(s) I. DOI: 10.1107/S1600536811025785/jh2306Isup2.hkl

Supplementary material file. DOI: 10.1107/S1600536811025785/jh2306Isup3.cml

Additional supplementary materials:  crystallographic information; 3D view; checkCIF report

## Figures and Tables

**Table 1:** Hydrogen-bond geometry (Å, °)

*D*—H⋯*A*	*D*—H	H⋯*A*	*D*⋯*A*	*D*—H⋯*A*
N1—H1*A*⋯S1^i^	0.87 (2)	2.54 (2)	3.380 (2)	163.6 (17)

Symmetry code: (i) 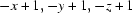.

**Fig. 1.**
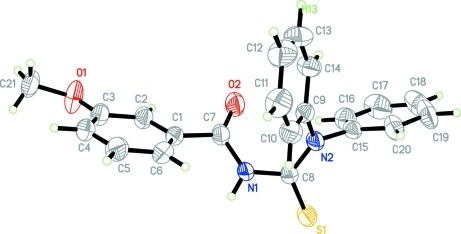
The molecular structure of the title compound with displacement ellipsoids drawn at the 50% probability level.

**Fig. 2.**
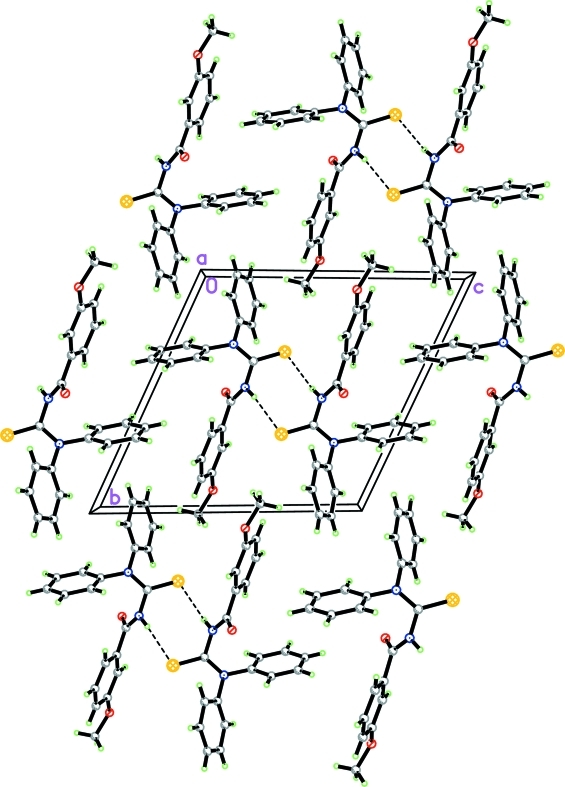
A packing diagram of the title compound viewed down the a-axis showing the intermolecular hydrogen bonds N1—H1···S1 (-x + 1, -y + 1, -z + 1) centrosymmetric dimers along a-axis.

## supplementary crystallographic information

### Comment

Benzoylthiourea compounds act as a chelating ligands by deprotonation of the amide group and coordinate *via* a thiolate form through the S and O atoms to form neutral complexes with cobalt (Weiqun *et al.*, 2005) and platinum (Circu *et al.*, 2009).

The title compound, I, is a thiourea derivative analogous to our previously reported compounds (Md Nasir *et al.*, 2011; Al-abbasi & Kassim, 2011). The thiono and the carbonyl groups are *trans* positioned with respect to N1—C8 bond with C7N1C8S1 torsion angle of -129.10 (16)°. The methoxy group is coplanar with the parent benzene ring with the largest deviation from the mean planes of (O1/C1/C2/C3/C4/C5/C6/C7) of -0.051 (2)° for C7 and the dihedral angle between the same plane and the thioamide mean planes (S1/N1/N2/C8) is 87.45 (8)°.

In the crystal structure, Intermolecular N1—H1A···S1 hydrogen bond link the molecules into a centrosymmetric dimers forming channel parallel to the *a*-axis.

### Experimental

The title compound was prepared according to a previously reported procedure (Al-abbasi *et al.*, 2010) using 3-methoxybenzoyl chloride and diphenylamine as the appropriate starting materials. A yellowish crystal, suitable for X-ray crystallography, was obtained by a slow evaporation from ethanol solution at room temperature (yield 75%).

### Refinement

Hydrogen atom of the amide group was determined from the difference Fourier map and N—H was initially fixed at 0.86(0.01) Å and allowed to be refined on the parent N atom with *U*_iso_(H) = 1.2*U*_eq_(N). All other H atoms were positioned geometrically with C—H bond lengths in the range 0.93 - 0.97 Å and refined in the riding model approximation with *U*_iso_(H)=1.2*U*_eq_(C,*N*), except for methyl group where *U*_iso_(H)= 1.5*U*_eq_(C).

### Crystal data

**Table:**

C_21_H_18_N_2_O_2_S	*Z* = 2
*M**_r_* = 362.43	*F*(000) = 380
Triclinic, *P*1	*D*_x_ = 1.308 Mg m^−^^3^
Hall symbol: -P 1	Melting point = 412.15–410.15 K
*a* = 6.056 (3) Å	Mo *K*α radiation, λ = 0.71073 Å
*b* = 12.895 (7) Å	Cell parameters from 3612 reflections
*c* = 13.344 (7) Å	θ = 6.4–55.2°
α = 112.796 (9)°	µ = 0.19 mm^−^^1^
β = 100.336 (10)°	*T* = 298 K
γ = 97.951 (10)°	Plate, yellow
*V* = 920.0 (8) Å^3^	0.52 × 0.23 × 0.03 mm

### Data collection

**Table:**

Bruker SMART APEX CCD area-detector diffractometer	3612 independent reflections
Radiation source: fine-focus sealed tube	2663 reflections with *I* > 2σ(*I*)
graphite	*R*_int_ = 0.029
ω scan	θ_max_ = 26.0°, θ_min_ = 1.7°
Absorption correction: multi-scan (*SADABS*; Bruker, 2000)	*h* = −7→7
*T*_min_ = 0.906, *T*_max_ = 0.994	*k* = −15→15
10189 measured reflections	*l* = −16→16

### Refinement

**Table:**

Refinement on *F*^2^	Primary atom site location: structure-invariant direct methods
Least-squares matrix: full	Secondary atom site location: difference Fourier map
*R*[*F*^2^ > 2σ(*F*^2^)] = 0.038	Hydrogen site location: inferred from neighbouring sites
*wR*(*F*^2^) = 0.110	H atoms treated by a mixture of independent and constrained refinement
*S* = 1.04	*w* = 1/[σ^2^(*F*_o_^2^) + (0.0578*P*)^2^ + 0.1546*P*] where *P* = (*F*_o_^2^ + 2*F*_c_^2^)/3
3612 reflections	(Δ/σ)_max_ < 0.001
240 parameters	Δρ_max_ = 0.17 e Å^−^^3^
0 restraints	Δρ_min_ = −0.27 e Å^−^^3^

### Special details

**Table:**

Geometry. All e.s.d.'s (except the e.s.d. in the dihedral angle between two l.s. planes) are estimated using the full covariance matrix. The cell e.s.d.'s are taken into account individually in the estimation of e.s.d.'s in distances, angles and torsion angles; correlations between e.s.d.'s in cell parameters are only used when they are defined by crystal symmetry. An approximate (isotropic) treatment of cell e.s.d.'s is used for estimating e.s.d.'s involving l.s. planes.
Refinement. Refinement of *F*^2^ against ALL reflections. The weighted *R*-factor *wR* and goodness of fit *S* are based on *F*^2^, conventional *R*-factors *R* are based on *F*, with *F* set to zero for negative *F*^2^. The threshold expression of *F*^2^ > σ(*F*^2^) is used only for calculating *R*-factors(gt) *etc*. and is not relevant to the choice of reflections for refinement. *R*-factors based on *F*^2^ are statistically about twice as large as those based on *F*, and *R*- factors based on ALL data will be even larger.

### Fractional atomic coordinates and isotropic or equivalent isotropic displacement parameters (Å^2^)

**Table:**

	*x*	*y*	*z*	*U*_iso_*/*U*_eq_	
S1	0.28578 (10)	0.32442 (4)	0.43411 (4)	0.05684 (19)	
O1	0.0958 (3)	0.92764 (11)	0.40494 (14)	0.0667 (4)	
O2	−0.0666 (2)	0.49768 (10)	0.27758 (12)	0.0554 (4)	
N1	0.2897 (3)	0.47668 (11)	0.34630 (12)	0.0403 (4)	
N2	0.0990 (2)	0.28974 (11)	0.22465 (10)	0.0372 (3)	
C6	0.4600 (3)	0.67429 (15)	0.30353 (15)	0.0458 (4)	
H6	0.5408	0.6164	0.2821	0.055*	
C5	0.5442 (3)	0.78217 (16)	0.30918 (17)	0.0504 (5)	
H5	0.6817	0.7962	0.2898	0.060*	
C4	0.4287 (3)	0.86899 (15)	0.34293 (15)	0.0458 (4)	
H4	0.4889	0.9413	0.3471	0.055*	
C3	0.2235 (3)	0.84874 (14)	0.37065 (15)	0.0438 (4)	
C2	0.1354 (3)	0.74050 (14)	0.36416 (14)	0.0430 (4)	
H2	−0.0031	0.7264	0.3827	0.052*	
C1	0.2519 (3)	0.65424 (13)	0.33049 (13)	0.0380 (4)	
C21	0.1482 (4)	1.03154 (16)	0.39139 (19)	0.0595 (5)	
H21A	0.2992	1.0754	0.4378	0.089*	
H21B	0.0366	1.0762	0.4132	0.089*	
H21C	0.1443	1.0132	0.3141	0.089*	
C7	0.1389 (3)	0.53717 (14)	0.31542 (14)	0.0405 (4)	
C8	0.2192 (3)	0.36345 (13)	0.33048 (13)	0.0376 (4)	
C9	0.1204 (3)	0.31150 (13)	0.12858 (13)	0.0369 (4)	
C14	−0.0742 (3)	0.29310 (16)	0.04667 (14)	0.0476 (4)	
H14	−0.2192	0.2659	0.0529	0.057*	
C13	−0.0526 (4)	0.31537 (19)	−0.04475 (16)	0.0610 (6)	
H13	−0.1836	0.3029	−0.1002	0.073*	
C12	0.1610 (4)	0.35575 (19)	−0.05447 (17)	0.0618 (6)	
H12	0.1745	0.3719	−0.1155	0.074*	
C11	0.3535 (4)	0.37199 (18)	0.02626 (18)	0.0579 (5)	
H11	0.4983	0.3987	0.0195	0.070*	
C10	0.3355 (3)	0.34923 (15)	0.11738 (15)	0.0464 (4)	
H10	0.4675	0.3592	0.1712	0.056*	
C15	−0.0664 (3)	0.18826 (13)	0.20578 (13)	0.0388 (4)	
C16	−0.2480 (3)	0.20071 (17)	0.25501 (16)	0.0523 (5)	
H16	−0.2582	0.2736	0.3035	0.063*	
C17	−0.4148 (4)	0.1046 (2)	0.2320 (2)	0.0697 (6)	
H17	−0.5374	0.1125	0.2654	0.084*	
C18	−0.4001 (4)	−0.0022 (2)	0.16034 (19)	0.0757 (8)	
H18	−0.5141	−0.0668	0.1442	0.091*	
C19	−0.2184 (4)	−0.01469 (17)	0.11210 (17)	0.0691 (7)	
H19	−0.2089	−0.0879	0.0640	0.083*	
C20	−0.0481 (4)	0.08113 (14)	0.13453 (14)	0.0505 (5)	
H20	0.0756	0.0729	0.1020	0.061*	
H1A	0.416 (3)	0.5170 (16)	0.3974 (17)	0.049 (5)*	

### Atomic displacement parameters (Å^2^)

**Table:**

	*U*^11^	*U*^22^	*U*^33^	*U*^12^	*U*^13^	*U*^23^
S1	0.0816 (4)	0.0375 (2)	0.0398 (3)	−0.0063 (2)	−0.0094 (2)	0.02171 (19)
O1	0.0816 (11)	0.0378 (7)	0.1021 (12)	0.0228 (7)	0.0492 (9)	0.0372 (7)
O2	0.0428 (8)	0.0374 (7)	0.0779 (9)	0.0029 (6)	0.0069 (7)	0.0217 (6)
N1	0.0461 (9)	0.0274 (7)	0.0387 (7)	−0.0018 (6)	−0.0051 (7)	0.0153 (6)
N2	0.0446 (8)	0.0302 (7)	0.0328 (7)	0.0003 (6)	0.0040 (6)	0.0142 (5)
C6	0.0488 (11)	0.0418 (9)	0.0496 (10)	0.0113 (8)	0.0086 (8)	0.0238 (8)
C5	0.0430 (10)	0.0521 (11)	0.0630 (12)	0.0058 (9)	0.0131 (9)	0.0333 (9)
C4	0.0488 (11)	0.0375 (9)	0.0517 (10)	0.0015 (8)	0.0055 (8)	0.0253 (8)
C3	0.0518 (11)	0.0335 (9)	0.0469 (9)	0.0085 (8)	0.0110 (8)	0.0188 (7)
C2	0.0461 (10)	0.0367 (9)	0.0495 (10)	0.0072 (8)	0.0132 (8)	0.0218 (8)
C1	0.0430 (10)	0.0328 (8)	0.0361 (8)	0.0026 (7)	0.0026 (7)	0.0176 (7)
C21	0.0784 (15)	0.0376 (10)	0.0712 (13)	0.0151 (10)	0.0196 (11)	0.0313 (10)
C7	0.0489 (11)	0.0310 (8)	0.0379 (8)	0.0046 (7)	0.0078 (7)	0.0137 (7)
C8	0.0401 (9)	0.0309 (8)	0.0372 (8)	0.0020 (7)	0.0029 (7)	0.0147 (7)
C9	0.0448 (10)	0.0315 (8)	0.0334 (8)	0.0053 (7)	0.0072 (7)	0.0152 (6)
C14	0.0459 (11)	0.0524 (10)	0.0402 (9)	0.0039 (8)	0.0045 (8)	0.0205 (8)
C13	0.0669 (14)	0.0732 (13)	0.0408 (10)	0.0146 (11)	0.0008 (9)	0.0283 (10)
C12	0.0808 (16)	0.0711 (14)	0.0466 (11)	0.0179 (12)	0.0230 (11)	0.0353 (10)
C11	0.0609 (13)	0.0614 (12)	0.0640 (12)	0.0120 (10)	0.0276 (10)	0.0350 (10)
C10	0.0463 (11)	0.0460 (10)	0.0472 (10)	0.0078 (8)	0.0090 (8)	0.0223 (8)
C15	0.0432 (10)	0.0341 (8)	0.0340 (8)	−0.0015 (7)	0.0017 (7)	0.0162 (7)
C16	0.0512 (12)	0.0517 (11)	0.0542 (11)	0.0064 (9)	0.0130 (9)	0.0247 (9)
C17	0.0547 (13)	0.0831 (16)	0.0705 (14)	−0.0078 (12)	0.0141 (11)	0.0403 (13)
C18	0.0862 (17)	0.0643 (14)	0.0560 (12)	−0.0330 (12)	0.0002 (12)	0.0286 (11)
C19	0.1047 (19)	0.0375 (10)	0.0451 (11)	−0.0142 (11)	0.0102 (12)	0.0110 (8)
C20	0.0684 (13)	0.0364 (9)	0.0386 (9)	0.0007 (9)	0.0121 (9)	0.0118 (7)

### Geometric parameters (Å, °)

**Table:**

S1—C8	1.6503 (17)	C21—H21C	0.9600
O1—C3	1.351 (2)	C9—C14	1.378 (2)
O1—C21	1.421 (2)	C9—C10	1.381 (3)
O2—C7	1.210 (2)	C14—C13	1.381 (3)
N1—C8	1.384 (2)	C14—H14	0.9300
N1—C7	1.387 (2)	C13—C12	1.373 (3)
N1—H1A	0.87 (2)	C13—H13	0.9300
N2—C8	1.352 (2)	C12—C11	1.368 (3)
N2—C9	1.438 (2)	C12—H12	0.9300
N2—C15	1.439 (2)	C11—C10	1.375 (3)
C6—C5	1.382 (2)	C11—H11	0.9300
C6—C1	1.389 (3)	C10—H10	0.9300
C6—H6	0.9300	C15—C20	1.374 (3)
C5—C4	1.373 (3)	C15—C16	1.376 (3)
C5—H5	0.9300	C16—C17	1.377 (3)
C4—C3	1.378 (3)	C16—H16	0.9300
C4—H4	0.9300	C17—C18	1.365 (4)
C3—C2	1.387 (2)	C17—H17	0.9300
C2—C1	1.371 (3)	C18—C19	1.369 (4)
C2—H2	0.9300	C18—H18	0.9300
C1—C7	1.489 (2)	C19—C20	1.389 (3)
C21—H21A	0.9600	C19—H19	0.9300
C21—H21B	0.9600	C20—H20	0.9300
			
C3—O1—C21	118.49 (16)	C14—C9—C10	119.92 (16)
C8—N1—C7	122.68 (15)	C14—C9—N2	119.86 (15)
C8—N1—H1A	116.5 (13)	C10—C9—N2	120.21 (15)
C7—N1—H1A	117.1 (13)	C9—C14—C13	119.56 (18)
C8—N2—C9	122.38 (13)	C9—C14—H14	120.2
C8—N2—C15	119.92 (13)	C13—C14—H14	120.2
C9—N2—C15	117.62 (12)	C12—C13—C14	120.52 (19)
C5—C6—C1	118.54 (17)	C12—C13—H13	119.7
C5—C6—H6	120.7	C14—C13—H13	119.7
C1—C6—H6	120.7	C11—C12—C13	119.54 (18)
C4—C5—C6	121.22 (18)	C11—C12—H12	120.2
C4—C5—H5	119.4	C13—C12—H12	120.2
C6—C5—H5	119.4	C12—C11—C10	120.76 (19)
C5—C4—C3	119.88 (16)	C12—C11—H11	119.6
C5—C4—H4	120.1	C10—C11—H11	119.6
C3—C4—H4	120.1	C11—C10—C9	119.66 (18)
O1—C3—C4	124.89 (15)	C11—C10—H10	120.2
O1—C3—C2	115.53 (16)	C9—C10—H10	120.2
C4—C3—C2	119.57 (17)	C20—C15—C16	120.88 (16)
C1—C2—C3	120.20 (17)	C20—C15—N2	119.82 (16)
C1—C2—H2	119.9	C16—C15—N2	119.20 (16)
C3—C2—H2	119.9	C15—C16—C17	119.6 (2)
C2—C1—C6	120.58 (15)	C15—C16—H16	120.2
C2—C1—C7	117.72 (16)	C17—C16—H16	120.2
C6—C1—C7	121.53 (16)	C18—C17—C16	120.1 (2)
O1—C21—H21A	109.5	C18—C17—H17	120.0
O1—C21—H21B	109.5	C16—C17—H17	120.0
H21A—C21—H21B	109.5	C17—C18—C19	120.30 (19)
O1—C21—H21C	109.5	C17—C18—H18	119.9
H21A—C21—H21C	109.5	C19—C18—H18	119.9
H21B—C21—H21C	109.5	C18—C19—C20	120.5 (2)
O2—C7—N1	123.01 (15)	C18—C19—H19	119.8
O2—C7—C1	122.65 (16)	C20—C19—H19	119.8
N1—C7—C1	114.33 (15)	C15—C20—C19	118.6 (2)
N2—C8—N1	114.68 (14)	C15—C20—H20	120.7
N2—C8—S1	124.04 (12)	C19—C20—H20	120.7
N1—C8—S1	121.26 (12)		

### Hydrogen-bond geometry (Å, °)

**Table:**

*D*—H···*A*	*D*—H	H···*A*	*D*···*A*	*D*—H···*A*
N1—H1A···S1^i^	0.87 (2)	2.54 (2)	3.380 (2)	163.6 (17)

Symmetry codes: (i) −*x*+1, −*y*+1, −*z*+1.
